# Frog Virus 3 Infection, Cultured American Bullfrogs

**DOI:** 10.3201/eid1302.061073

**Published:** 2007-02

**Authors:** Debra L. Miller, Sreekumari Rajeev, Matthew J. Gray, Charles A. Baldwin

**Affiliations:** * University of Georgia, Tifton, Georgia, USA; † University of Tennessee, Knoxville, Tennessee, USA

**Keywords:** *Aeromonas hydrophila*, aquaculture, bullfrog, FV3, metamorphs, *Rana catesbeiana*, letter

**To the Editor:** Ranaculture, the practice of farm-raising frogs for scientific and culinary purposes, is practiced in many countries, including the United States (*1*). As with aquaculture, most ranaculture challenges relate to husbandry and disease. In aquaculture, iridovirus infections are reportable and can result in large-scale fish deaths (*2*,*3*). The family *Iridoviridae* is composed of *Iridovirus*, *Chloriridovirus*, *Ranavirus*, and *Lymphocystivirus.* The first 2 infect insects; the latter 2, lower vertebrates (*4*). Infection with frog virus 3 (FV3), the type species of the genus *Ranavirus,* results in edema, hemorrhage, and necrosis of lymphoid tissue, hematopoietic tissue, liver, spleen, and renal tubules (*3*,*5*); mortality rates in free-ranging amphibians are >90% (*6*).

In May 2006, a commercial American bullfrog (*Rana catesbeiana*) ranaculture facility suffered massive (>50%) deaths of frogs that had recently undergone metamorphosis. The facility, with >25 years of experience, uses an on-site breeding colony and an all-in, all-out system, in which cohorts are moved through the system as 1 unit. Well water is used throughout. The breeding colony and larvae are housed in outdoor tanks to expose them to ambient climatic conditions, thought to facilitate breeding and development. Outdoor tanks are covered with mesh to prevent predation by birds. After metamorphosis, animals are moved indoors.

Full necropsies were performed on 3 of the recent metamorphs. A set of fixed tissue sections from all organs was routinely processed for light microscopic examination. An identical set of fresh tissue sections was collected for routine bacterial culture and viral analysis. Bacterial isolates were speciated by using an automated system (Sensititer, Trek Diagnostic Systems, Westlake, OH, USA) or conventional biochemical testing. Virus isolation was performed by using a variety of cell lines; random isolates were verified by electron microscopy. A heminested PCR targeting the major capsid protein gene was performed (*3*), amplicons were sequenced (SeqWright DNA Technology Services, Houston, TX, USA), and a GenBank BLAST search (www.ncbi.nlm.nih.gov/Genbank/) was performed.

Pathologic changes in all metamorphs were similar. Gross findings were as follows: irregular gray patches on the skin, cutaneous and enteric erythema, mottled heart and kidneys, pale and friable livers, and enlarged gall bladders. Histologic examination showed lymphoid depletion and necrosis in the thymus and other lymphoid tissues and necrosis in the liver, spleen (Figure), and epidermis. Scattered intracytoplasmic inclusion bodies were observed in the spleen (Figure B inset). Epithelial degeneration was noted in the renal tubules. Bacteria were present within the dermal lesions, glomerular tufts and vessels of the kidney, and, rarely, in the spleen and sinusoids of the liver.

**Figure:**
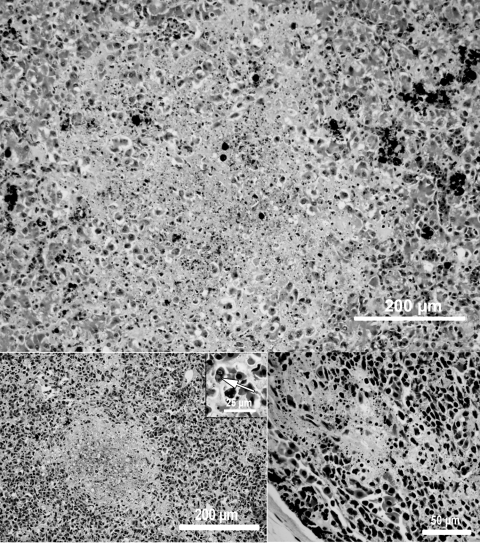
Light microscopic images of the liver (A), spleen (B), and thymus (C) showing necrosis in an American bullfrog (*Rana catesbeiana*) metamorph infected with frog virus 3. Spleen (B) inset shows intracytoplasmic viral inclusion bodies. Hematoxylin and eosin stain.

Iridovirus was isolated and confirmed by PCR. A BLAST search of the resulting sequence (GenBank accession no. EF101698) showed identity with FV3. *Aeromonas hydrophila* was cultured from the internal organs and dermal lesions. Diagnosis was FV3 infection with a secondary *A. hydrophila* infection. Incidentally, 5 larval bullfrogs obtained from this cohort ≈6 months earlier died of nitrate toxicity 1 month after acquisition. PCR and sequencing identified FV3 (GenBank accession no. EF101697) in these 5 larvae; secondary bacterial invasion was absent.

Susceptibility to FV3 is thought to vary by species and life cycle (*5*–*7*). How the amphibian immune system responds to FV3 infection is not known. Critical periods for infectivity likely include the time before the larval immune system develops, at metamorphosis while the larval immune system is being dismantled, and during periods of exogenous stressors (e.g., movement of the animals from outside to inside tanks). Consequently, we hypothesize that the stress of recent metamorphosis, along with the added stress of movement from outside to inside, likely increased the frogs’ susceptibility to FV3.

Further, with lymphoid depletion and multiorgan compromise (necrosis), individual frogs become susceptible to opportunistic pathogens, such as *A. hydrophila*, especially when the innate immune system fails (i.e., skin abrasions). *A. hydrophila* infections alone can result in considerable loss in ranaculture systems (*8*). Unfortunately, specimens often are submitted for bacterial analysis only, not viral testing. Thus, the effects of *Ranavirus* on ranaculture remain unknown. As with free-ranging populations, in which coinfections have been reported (*9*), ranaculture populations that had a diagnosis of *A. hydrophila* may have had an underlying *Ranavirus* infection.

In vertebrates, iridovirus is thought to be transmitted only horizontally (*10*). This ranaculture facility kept frogs separated according to size, to decrease cannibalism. Possible routes of FV3 exposure in this facility are the following: exposure of the larval tank to an infected free-ranging frog, mechanical transmission during routine husbandry, or mechanical transmission by insects. Regardless, at this time the frogs can be treated only for bacterial pathogens; however, early detection and reduction of exogenous stressors may help less-affected bullfrogs clear the virus (*11*) and ultimately reduce loss.

All-in, all-out ranaculture systems may be able to eliminate FV3 infection by preventing exposure of cultured larvae to mechanical vectors and native anurans. Ranaculture systems must eliminate this virus before translocating infected frogs to naive systems. Because amphibians are declining globally, exposure of captive wildlife to free-ranging populations should be minimized.

## References

[1] Miles J, Williams J, Hailey A. Frog farming. Investigation of biological and mechanical agents to increase the consumption of pelleted food by adult *Rana temporaria.* Applied Herpetology. 2004;1:271–86. 10.1163/157075403323012223

[2] Ahne W, Bremont M, Hedrick RP, Hyatt AD, Whittington RJ. Iridoviruses associated with epizootic haematopoietic necrosis (EHN) in aquaculture. World J Microbiol Biotechnol. 1997;13:367–73. 10.1023/A:1018563930712

[3] Kattenbelt JA, Hyatt AD, Gould AR. Recovery of ranavirus dsDNA from formalin-fixed archival material. Dis Aquat Organ. 2000;39:151–4. 10.3354/dao039151 10715821

[4] Tan WGH, Barkman TJ, Chinchar VG, Essani K. Comparative genomic analyses of frog virus 3, type species of the genus *Ranavirus* (family *Iridoviridae*). Virology. 2004;323:70–84. 10.1016/j.virol.2004.02.019 15165820

[5] Robert J, Morales H, Buck W, Cohen N, Marr S, Gantress J. Adaptive immunity and histopathology in frog virus 3–infected *Xenopus.* Virology. 2005;332:667–75. 10.1016/j.virol.2004.12.012 15680432

[6] Daszak P, Berger L, Cunningham AA, Hyatt AD, Green DE, Speare R. Emerging infectious diseases and amphibian population declines. Emerg Infect Dis. 1999;5:735–48. 10603206 10.3201/eid0506.990601 PMC2640803

[7] Greer AL, Berrill M, Wilson PJ. Five amphibian mortality events associated with *Ranavirus* infection in south central Ontario, Canada. Dis Aquat Organ. 2005;67:9–14. 10.3354/dao067009 16385802

[8] Mauel MJ, Miller DL, Frazier KS, Hines ME II. Bacterial pathogens isolated from cultured bullfrogs (*Rana castesbeiana*). J Vet Diagn Invest. 2002;14:431–3. 12296400 10.1177/104063870201400515

[9] Cunningham AA, Langton TES, Bennett PM, Lewin JF, Drury SEN, Gough RE, Pathological and microbiological findings from incidents of unusual mortality of the common frog (*Rana temporaria*). Philos Trans R Soc Lond B Biol Sci. 1996;351:1539–57. 10.1098/rstb.1996.0140 8962441

[10] Hunter W, Sinisterra XH, McKenxie CL, Shatters RG. Iridovirus infection and vertical transmission in citrus aphids. Proceedings of the Annual Meeting of the Florida State Horticultural Society; Stuart (FL): 2001 Jun 10–12. 2001;114:70–2.

[11] Green DE, Converse KA, Schrader AK. Epizootiology of sixty-four amphibian morbidity and mortality events in the USA, 1996–2001. Ann N Y Acad Sci. 2002;969:323–39. 12381613 10.1111/j.1749-6632.2002.tb04400.x

